# Phenological mismatch affects individual fitness and population growth in the winter moth

**DOI:** 10.1098/rspb.2023.0414

**Published:** 2023-08-30

**Authors:** Natalie E. van Dis, Geert-Jan Sieperda, Vidisha Bansal, Bart van Lith, Bregje Wertheim, Marcel E. Visser

**Affiliations:** ^1^ Department of Animal Ecology, Netherlands Institute of Ecology (NIOO-KNAW), PO Box 50, 6700 AB Wageningen, The Netherlands; ^2^ Groningen Institute for Evolutionary Life Sciences, University of Groningen, 9747 AG Groningen, The Netherlands

**Keywords:** climate change, phenological mismatch, demography, fitness, population cycles, *Operophtera brumata*

## Abstract

Climate change can severely impact species that depend on temporary resources by inducing phenological mismatches between consumer and resource seasonal timing. In the winter moth, warmer winters caused eggs to hatch before their food source, young oak leaves, became available. This phenological mismatch changed the selection on the temperature sensitivity of egg development rate. However, we know little about the fine-scale fitness consequences of phenological mismatch at the individual level and how this mismatch affects population dynamics in the winter moth. To determine the fitness consequences of mistimed egg hatching relative to timing of oak budburst, we quantified survival and pupation weight in a feeding experiment. We found that mismatch greatly increased mortality rates of freshly hatched caterpillars, as well as affecting caterpillar growth and development time. We then investigated whether these individual fitness consequences have population-level impacts by estimating the effect of phenological mismatch on population dynamics, using our long-term data (1994–2021) on relative winter moth population densities at four locations in The Netherlands. We found a significant effect of mismatch on population density with higher population growth rates in years with a smaller phenological mismatch. Our results indicate that climate change-induced phenological mismatch can incur severe individual fitness consequences that can impact population density in the wild.

## 1. Introduction

Climate change has led to seasonal timing shifts in a large range of species [1,2]. Interacting species often do not shift at the same rate leading to the occurrence of phenological mismatches: a mismatch between the timing of a species' resource demands and the timing of its resource abundance [3,4]. Mismatches can have severe consequences for individuals' fitness, leading to decreased survival and reproduction with potential long-term consequences for population viability [4]. To understand how populations will respond to climate change-induced phenological mismatch, we need to understand the link between individual fitness effects and population demography [5,6].

Fitness consequences of climate change-induced phenological mismatch lead to phenotypic selection, such as selection for earlier breeding in songbirds [7,8] and earlier flowering time in plants [9]. As survival and/or reproduction are affected, this selection is expected to influence population growth rates. For example, climate change-induced mismatches between the timing of reproduction and timing of plant growth have led to reduced demographic rates in roe deer and arctic breeding geese [10,11]. Nevertheless, fitness consequences at the individual level and their effects on the population level are not always straightforward due to density dependence and environmental stochasticity [4,6]. In a small songbird, climate change-induced directional selection at the egg-laying stage in spring is counterbalanced by improved winter survival in low-density years [12]. This example illustrates that population growth rates are ultimately determined by the absolute performance of individuals over the year. However, few studies have linked the effect of mismatch-induced selection to demography rates [4], so far showing that these two processes are often uncoupled [12–14].

The winter moth, *Operophtera brumata*, is a classic model species to investigate population dynamics [15]. Interestingly, it is also one of the few species that was found to have genetically adapted in response to selection from climate change [16,17]. Warmer winters induced a severe phenological mismatch with winter moth eggs hatching more than 10 days before the timing of oak, *Quercus robur*, budburst [18]. Over just a decade of this directional selection, the temperature response of winter moth egg development rate genetically changed such that eggs are now better timed to their food source [17].

Early work on the population dynamics of winter moths indicated that although phenological mismatch could have a pronounced effect on winter disappearance (i.e. the difference between adult population size in winter and fully grown caterpillar population size in the following spring), it did not influence adult winter moth densities between years [15]. Winter moth populations throughout northern Europe show characteristic cyclic dynamics with 9–11 year cycles [19], which is a common phenomenon in forest Lepidoptera [20]. In the winter moth, these cycles are thought to be mainly driven by two density-dependent mechanisms at the pupal stage: density-dependent regulation by generalist predators (e.g. beetles and small mammals) in combination with delayed density-dependent pupal parasitism by a specialist parasitoid [15]. Nevertheless, the magnitude of climate change-induced phenological mismatch as well as its continued directional selection over more than 10 years [17] might be conditions under which the fitness consequences of this selection can influence population growth rates [6]. Experimental work showed that a mismatch of 5 days too late or too early relative to oak budburst can have severe fitness consequences for winter moth caterpillars in terms of both survival and fecundity [21]. However, the exact shape of the fitness curve of phenological mismatch in the winter moth as well as the link with its population dynamics are still unknown.

Here, we investigated the fitness consequences of phenological mismatch at the individual level and how these mismatch consequences translate to the population level. In a phenological mismatch experiment, we manipulated the hatching of eggs collected from wild winter moth females to hatch 1–5 days before or after the timing of oak budburst. We then used the observed survival rates and pupation weights (as a proxy of fecundity) to construct a fine-scale fitness curve for phenological mismatch. Next, we modelled the cyclic population dynamics observed in our long-term data (1994–2021) on relative winter moth population densities at four locations in The Netherlands, and investigated how much variance in population growth can be explained by phenological mismatch. From previous work (e.g. [21,22]), we expected that hatching before oak budburst would lead to increased mortality rates, while hatching after budburst would lead to decreased fecundity. As freshly hatched caterpillars are very sensitive to starvation [22], we expected that strong mismatch observed in the field would have affected population growth rates. However, as winter moth population dynamics seem to be mostly regulated at the pupal stage [15], we expected phenological mismatch to explain only a small portion of the variation. Connecting the individual fitness consequences of phenological mismatch to population demography will be instrumental in understanding the winter moth's response to climate change.

## 2. Material and methods

### (a) Long-term field data

**Figure 1.**
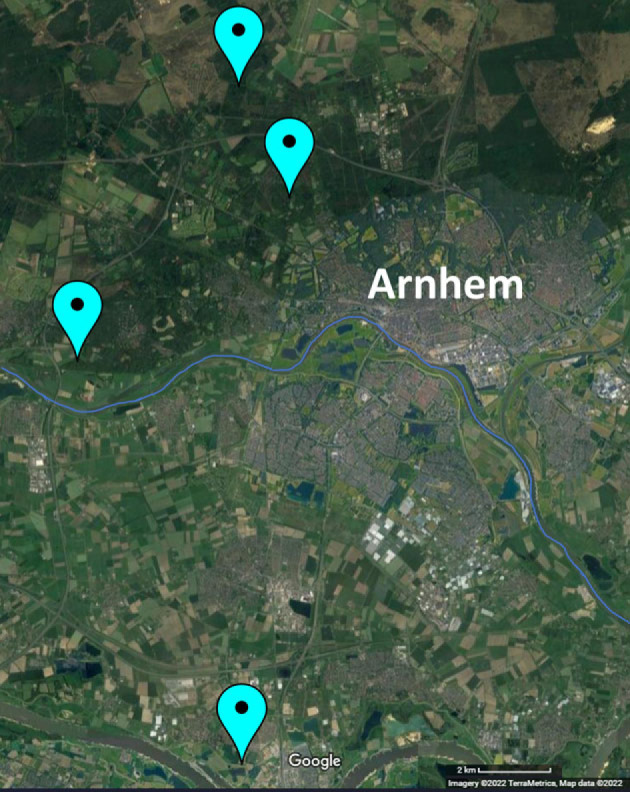
Locations of forests used for long-term field monitoring of winter moths in The Netherlands. Field data on winter moths were collected yearly since 1994 in four forests around the city of Arnhem, with from top to bottom: Hoge Veluwe (HV; 52°05′ N, 05°48′ E), Warnsborn (WA; 52°05′ N, 05°50′ E), Doorwerth (DO; 51°59′ N, 05°48′ E), and Oosterhout (OH; 51°55′ N, 05°50′ E) [17]. The river Rhine has been highlighted with a blue line.

Field data on winter moths were collected yearly since 1994 in four forests around Arnhem, The Netherlands (figure 1; Doorwerth (DO), Hoge Veluwe (HV), Oosterhout (OH) and Warnsborn (WA)) [17]. From the first week of November, insect traps were placed on multiple oak trees in each forest (DO: 7–15 trees, mean = 12 trees; HV: 12–24 trees, mean = 14 trees; OH: 6–36 trees, mean = 23 trees; WA: 4–19 trees, mean = 12 trees), aiming to use the same trees each year. Each trap was emptied twice a week, noting down the number of adults caught. For each catch date, at least three females from each trap were kept in an outdoor field shed, placed individually in a plastic container with a roll of tissue paper for laying eggs. If available, females were paired with a male caught on the same day from the same or a nearby trap. In early January, eggs from each female were counted and placed back in the field shed until hatching. From the start of March, hatching was monitored twice a week. Hatch date for each clutch of eggs was calculated as the day at which 50% of the clutch had hatched (D_50_). In the field, budburst of the oak trees on which winter moths were caught was monitored twice a week. The date of budburst was determined as the date that on average the buds in the crown of the tree reached the stage where young leaves started protruding [18,23]. For each clutch, phenological mismatch was calculated as the difference between oak budburst date and clutch D_50_ in days. To get the mean phenological mismatch per forest, we first calculated the mean phenological mismatch per catch tree, and then averaged the tree means to control for differences in sample size per tree. Similarly, we obtained the mean standard deviation of mismatch per forest as a measure of variance in mismatch.

### (b) Phenological mismatch experiment

In the spring of 2021, we conducted a phenological mismatch experiment with eggs collected from wild females caught as part of our long-term field monitoring (see above). In a split-brood experiment, timing of egg hatching was manipulated in climate cabinets to induce a seasonal timing mismatch with oak budburst (see below for further details), leading to hatching before (0–4 days) or after budburst (1–5 days), similar to a previous experiment [21]. As we manipulated phenological mismatch by changing hatch dates, the experiment included two photoperiod treatments to control for the effect of day length on winter moth developmental timing [24]. Caterpillars were given either a constant photoperiod, using the natural day length from the day of oak budburst (see below), or a naturally changing photoperiod with gradually incrementing photoperiods to mimic the increasing day length during spring. This photoperiod treatment allowed us to test for the effect of phenological mismatch on developmental timing. Specifically, individuals in the constant photoperiod treatment all received a day length associated with spring, but only received additional seasonal information through food quality and not through day length despite hatching on different days (i.e. comparing different mismatch treatments).

Starting in early spring when winter moth eggs were close to hatching (26 March 2021), clutches from 47 wild females caught at our long-term field sites (16 November–10 December) were split into 11–13 sub-clutches of at least 15 eggs and placed in climate cabinets at a constant 2°C to delay egg hatching. In the field, we monitored 86 mature European oak trees growing in a patch near the research institute for budburst three times a week (location: 51°59′53.43″ N, 5°39′44.13″ E). The date of budburst was determined in the same way as we did for the long-term monitored trees (see above). We selected 11 trees for the feeding experiment, which each had their budburst on the same day (3 May).

As soon as the tree buds in the field began to swell, every day one sub-clutch from each female was moved to a constant 20°C to induce staggered egg hatching. The number of hatching caterpillars was scored daily and six caterpillars from each sub-clutch were transferred to individual containers for treatment, randomly divided over the constant and changing photoperiod conditions (*N* = 3 replicates per clutch per treatment). Only clutches for which at least six eggs per day hatched were included in the experiment (*N* = 22 clutches, Areas: DO = 3 females, HV = 6 females, OH = 6 females, WA = 7 females). The three replicates per clutch were kept in separate climate cabinets at a constant temperature of 12.5°C (15°C on the first day of the experiment), with high humidity (70–95%) and a day–night light cycle that depended on the photoperiod treatment (Constant: photoperiod matching natural day length on 3 May; Changing: photoperiod changed three times a week to match the outside day length).

The experiment lasted for a period of 11 days from 29 April to 8 May, with oak budburst on 3 May. Caterpillars that hatched before budburst experienced a period of starvation (0–4 days) before they were fed for the first time, while caterpillars that hatched after budburst (1–5 days) were fed on the day of hatching with leaves harvested on that day. In total, the experiment included daily mismatch treatments for the changing photoperiod treatment (11 days) and every other day mismatch treatments for the constant photoperiod treatment (5 days). From thereon, three times a week for the duration of seven weeks, caterpillars were fed and their containers were cleaned. Caterpillars were fed with buds collected from the 11 oak trees that had their budburst on 3 May (see above). On feeding days, leaves were harvested in the morning, mixed together, and distributed randomly over the caterpillars to exclude the effect of individual host tree quality; and to take into account within-tree and between-tree variation that can be present despite trees having the same budburst date [25]. The progression of oak leaf development during the feeding period is depicted in electronic supplementary material, figure S1.1.

From 21 days after the start of the experiment, caterpillars were checked for survival and pupation three times a week. On the day of pupation (i.e. the first day the caterpillar initiated cocoon formation), each caterpillar was weighed and transferred to a falcon tube containing vermiculite (artificial soil). Pupae were kept in climate cabinets in darkness, with high humidity and at temperatures that mimicked natural soil temperatures at 10 cm depth (monthly averages of 2010–2020, obtained from Royal Dutch Meteorological Institute (KNMI) weather station De Bilt). From early November onwards, they were monitored for adult eclosion.

### (c) Statistical analysis

#### (i) Phenological mismatch experiment

We used the survival and pupation weight measures per caterpillar to estimate the fitness curve of phenological mismatch. Pupation weight was used as a proxy for fecundity as previously done for the winter moth [21]. Data were analysed in R v4.1.2 [26] at a significance level of *α* = 0.05, using mixed models with R package lme4 v1.1-28 [27]. For survival, we used a binomial mixed model with photoperiod treatment and mismatch treatment in days as fixed effects. We also included mismatch treatment squared as we expected a nonlinear relationship from previous work [21], as well as the interaction between mismatch treatment and mismatch treatment squared with photoperiod treatment. MotherID was included as a random effect. For pupation weight, we used a linear mixed model including the same fixed and random effects as the survival model. Non-significant interaction terms (*p* > 0.05) were removed from the final models to be able to then test for main treatment effects. If there was no evidence for a nonlinear relationship (i.e. *p* > 0.05), we also removed mismatch treatment squared from the final models.

Previous work suggested that changes in leaf quality as a result of phenological mismatch might affect larval and pupal development times, as well as egg development time of the next generation through maternal effects [24,28]. We analysed larval development time in days with a linear mixed model using the same procedure and the same fixed and random effects as the fitness consequences models. Unfortunately, we could not analyse pupal development times since only a small proportion of the individuals survived until adulthood (14 out of 346 caterpillars that pupated) due to a major fungal infection in the pupae. Because we have never observed pupal fungal infection in previous experiments using the same protocol [24,28] and because pupae in different incubators and from different treatment groups were affected equally (electronic supplementary material, figure S1.2), we think the fungal infection was most likely due to contamination of the vermiculite substrate that the pupae were kept in, paired with the maintained high humidity.

To estimate the fitness curve of phenological mismatch, we used the predicted values from the final models for survival and pupation weight for each MotherID, excluding the effect of photoperiod treatment. We did not include larval development time, because it is unclear how it contributes to relative fitness compared to the major contributions of survival and pupation weight in the winter moth [29,30]. Absolute fitness was calculated by multiplying the predicted survival probabilities with the predicted pupation weights (mg) for each clutch and mismatch treatment, and the resulting fitness curve was expressed relative to the mismatch treatment day for which the winter moths had the highest fitness.

#### (ii) Cyclic population dynamics model

The raw long-term time series of winter moth densities indicated the presence of cyclic population dynamics. We assessed the presence of cyclic dynamics in R, using the autocorrelation function (ACF) and spectral density analysis [31,32] on ln-transformed female numbers per area, corrected for trapping effort. We chose to only look at female numbers, as ultimately females produce the next generation. Moreover, as winter moth females are flightless, we expected them to give a good estimate of local abundances. Time series from each area were checked for stationarity with the Kwiatkowski-Phillips-Schmidt-Shin (KPSS) test from R package tseries v0.10-51 [33] and did not need detrending.

To assess how much of the variance in winter moth population growth could be explained by phenological mismatch, we modelled population growth using linear feedback structures as fixed effects to account for the observed cyclic dynamics, and then estimated the effect of mismatch on the remaining variance in population growth rates. To select the order of linear feedback processes, we analysed the ln-transformed female numbers per area with autoregressive (AR) models [32] using Akaike's information criterion corrected for small sample sizes (AICc) for model selection with R package forecast v8.17 [34]; as well as using the partial rate correlation function (PRCF [35]) scripted in R (see deposited R scripts [36]).

Population dynamics were modelled in R using linear models, with the realized *per capita* rate of change (*R*) as our response variable, calculated as *R* = ln(N_t_/N_t__−1_), where N_t_ is the number of females observed in Year t [37]. The full model contained linear feedback structures up to and including the fourth order [ln(N_t– [1-4]_)] as fixed effects to model the cyclicity in population numbers; and the mean phenological mismatch per area as fixed effect to test for the effect of mismatch on the remaining variance in population growth rates. We furthermore included interaction terms between the feedback structures and area to account for potential differences in drivers of cyclicity; the standard deviation of phenological mismatch per area to account for between-tree and between-clutch variation in timing [25]; and the average number of eggs laid per female in the previous year (N_t__−1_) to account for changes in fecundity that could influence population growth [20]. Model residuals were checked for normality. To simplify the model, we first removed non-significant interaction terms (*p* > 0.05), followed by the removal of the non-significant covariates: number of eggs and the standard deviation of phenological mismatch. This way, we optimized the number of observations included in our final model for testing the variable of interest: the effect of phenological mismatch on population growth (*N* = 80 observations, 14–24 years per Area included). We used R package effectsize v0.7 [38] to calculate the proportion of variance explained by each parameter in the final model.

## 3. Results

### (a) Phenological mismatch experiment

**Figure 2.**
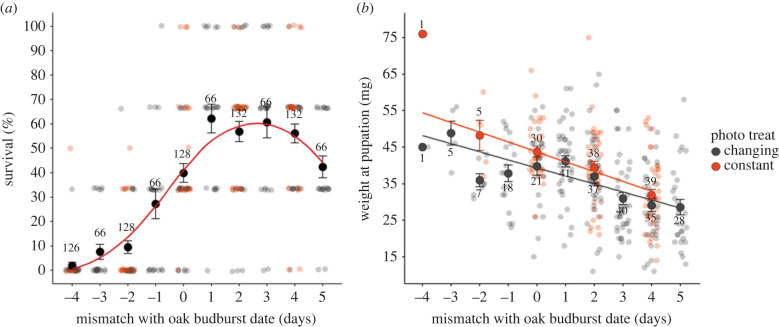
Fitness consequences of individual phenological mismatch. Mean ± s.e. survival percentages (*a*) and pupation weights (*b*) are shown for each mismatch treatment, with oak budburst on day 0. Numbers indicate the number of caterpillars per treatment group, with the raw data for each clutch (*a*) or individual caterpillar (*b*) depicted in the background. Sample points are coloured by photoperiod treatment, with lines giving the model fit for phenological mismatch. Means and model fit (red line) for survival have been averaged across the two photoperiods (*a*) as there was no effect of photoperiod treatment on survival percentages (estimate = −0.09 ± 0.16, *p* = 0.60; electronic supplementary material, table S1.1). There was an effect of photoperiod on pupation weight (estimate = 3.30 ± 1.14, *p* = 0.004; electronic supplementary material, table S1.2), which is why means and model fit for each photoperiod and mismatch treatment combination are plotted separately for pupation weight (*b*).

As expected, phenological mismatch between winter moth egg hatching and oak budburst affected survival rates and pupation weight of caterpillars (figure 2). The effect of phenological mismatch on survival was nonlinear, with a significant effect of mismatch (estimate = −1.70 ± 0.19, *p* < 0.001) as well as of mismatch squared (estimate = 0.11 ± 0.01, *p* < 0.001; electronic supplementary material, table S1.1). Caterpillars that hatched on the day of budburst were still prone to mortality, with on average 40% surviving. As caterpillars hatched earlier before budburst, survival steeply declined with only two caterpillars out of 126 surviving after 4 days of starvation (figure 2*a*). By contrast, hatching after budburst increased survival rates to 60%, but dropped off again to 40% for caterpillars that hatched 5 days after budburst. The effect of phenological mismatch on survival was not modulated by photoperiod treatment (interaction terms *p* > 0.05), nor was survival affected by photoperiod (estimate = −0.09 ± 0.16, *p* = 0.60; electronic supplementary material, table S1.1).

Phenological mismatch significantly affected weight at pupation (estimate = −2.51 ± 0.27, *p* < 0.001; electronic supplementary material, table S1.2), with caterpillars that could start feeding on younger oak leaves having higher pupation weights compared to caterpillars that hatched after budburst (figure 2*b*). This linear effect of phenological mismatch on pupation weight was not modulated by photoperiod (interaction terms *p* > 0.05), but there was a main photoperiod treatment effect (estimate = 3.30 ± 1.14, *p* = 0.004; electronic supplementary material, table S1.2). Caterpillars in the constant photoperiod treatment on average had higher pupation weights (figure 2*b*). Mismatch and photoperiod treatment effects were still present when excluding mismatch treatment −4 days (*N* = 2 caterpillars).

**Figure 3.**
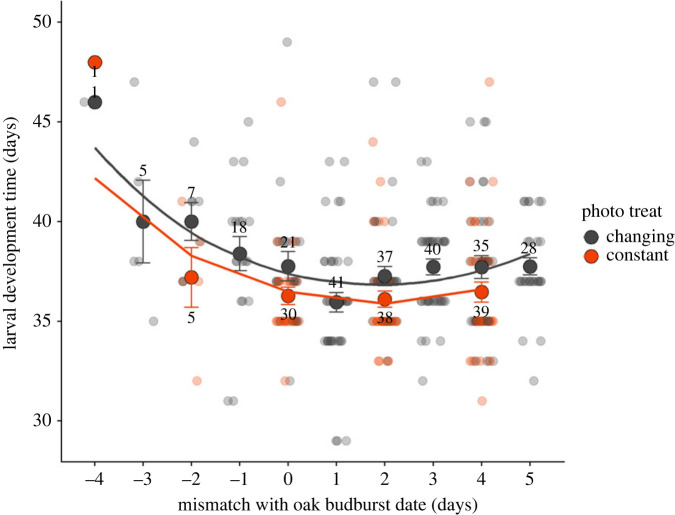
Effect of phenological mismatch on larval development time. Mean ± s.e. larval development time in days is shown for each mismatch treatment, with oak budburst on day 0. Numbers indicate the sample size per treatment group, with the raw data for each individual caterpillar depicted in the background. Sample points are coloured by photoperiod treatment, with lines giving the model fit for phenological mismatch. Means for each photoperiod and mismatch treatment combination are plotted separately, since there was a significant main effect of photoperiod treatment on larval development time (estimate = −0.95 ± 0.33, *p* = 0.004; electronic supplementary material, table S1.3). Tukey *post* *hoc* tests showed that both hatching before (Mismatch < 1) and hatching after the fitness peak (Mismatch > 2, figure 4) significantly increased larval development time compared to hatching on the days when fitness was highest (Mismatch = 1–2, *p* = 0.002 and *p* = 0.03 respectively).

Phenological mismatch significantly affected the length of larval development time (figure 3), showing a nonlinear effect (mismatch: estimate = −2.27 ± 0.44, *p* < 0.001; mismatch squared: estimate = 0.16 ± 0.03, *p* < 0.001; electronic supplementary material, table S1.3). Caterpillars that hatched close to the day of maximum fitness (mismatch day +2, see below) had the shortest larval development times, while larval development time increased for caterpillars that hatched earlier or later. The length of larval development time was also significantly affected by photoperiod treatment (estimate = −0.95 ± 0.33, *p* = 0.004; electronic supplementary material, table S1.3), with caterpillars from the constant photoperiod treatment on average pupating earlier than caterpillars in the changing photoperiod treatment (figure 3). Of the few caterpillars that reached adulthood (14 out of 346 pupated caterpillars), caterpillars from the constant photoperiod treatments tended to have longer pupal development times but this was not tested due to the small sample size (electronic supplementary material, figure S1.2a). The sex ratio of the small number of adults that eclosed showed no signs of deviating between treatments (electronic supplementary material, figure S1.2b).

**Figure 4.**
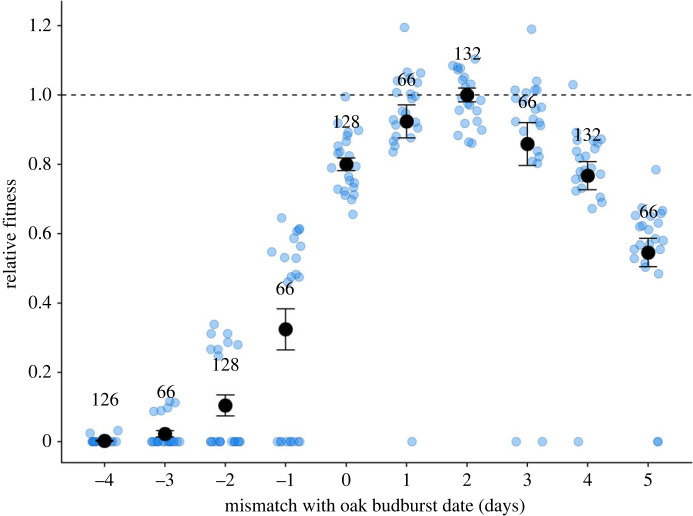
Relative fitness curve for individual phenological mismatch. Mean ± s.e. relative fitness for each mismatch treatment is shown, with oak budburst on day 0. Fitness was calculated by multiplying for each clutch the predicted survival probabilities with the predicted pupation weights for each mismatch treatment (figure 2), excluding the effect of photoperiod. Fitness is expressed relative to the highest fitness observed for caterpillars that hatched 2 days after oak budburst (dotted line). Numbers indicate the number of caterpillars that the fitness measures are based on, with sample points in blue depicting the fitness values for each clutch.

Figure 4 depicts the relative fitness curve for phenological mismatch, combining the predicted survival probabilities and the predicted pupation weights (as a proxy for fecundity) for each phenological mismatch treatment (figure 2). Fitness was maximized in caterpillars that hatched 2 days after budburst. The severe mortality rates of hatching too early led to a steep fitness decline, approaching zero for eggs hatching four days before budburst. The mean fitness loss for hatching earlier than the fitness peak was on average 14% per day, with rates up to 32% fitness loss per day for caterpillars that experienced starvation (figure 4). Hatching later than the fitness peak also led to fitness declines due to lower acquired pupal weights of on average 6% per day, up to 24% per day by mismatch treatment +5 days.

### (b) Cyclic population dynamics model

**Figure 5.**
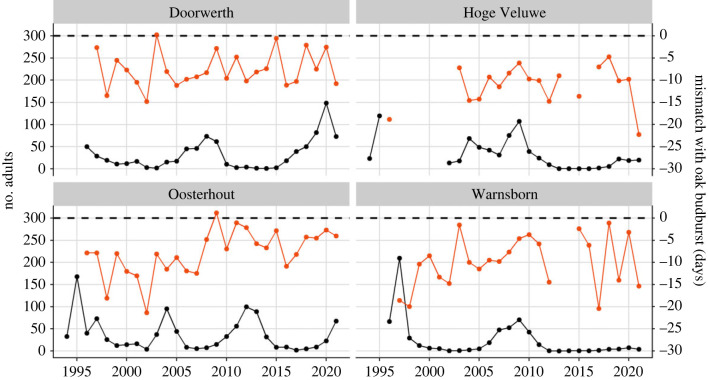
Winter moth population dynamics and population phenological mismatch at four locations in The Netherlands. Raw yearly winter moth densities (black points) and mean phenological mismatch (orange points) collected as part of a long-term monitoring scheme (1994–2021) are depicted per area (22–28 years of data per area). The dotted line indicates a mismatch of 0 days, i.e. the mean day of oak budburst in that area. Monitoring was interrupted at the Hoge Veluwe between 1995 and 2002 (6 years data gap). Data gaps in mismatch measures at the Hoge Veluwe and Warnsborn result from years in which traps were placed but no adults were caught.

Winter moth population densities at four locations in The Netherlands showed cyclic dynamics (figure 5), with female abundances showing the same pattern as the total number of adults (electronic supplementary material, figure S2.1). Cyclic dynamics were confirmed by ACF analysis and spectral density analysis (electronic supplementary material, figures S2.2 and S2.3). Spectral density peaks indicated 13.5-year cycles in winter moth densities at Doorwerth and Warnsborn, while population cycles at Oosterhout had a period of 10 years (electronic supplementary material, figure S2.3). The time series of the Hoge Veluwe population was too short to determine the cycling period due to a data gap of 6 years (figure 5).

The best feedback order structure to describe the observed population cycling differed between areas, but never exceeded four orders (AR models: table 1; PRCF: electronic supplementary material, figure S2.4). Population cycles at Doorwerth and Oosterhout were best described by and up to a four-order feedback structure as indicated by the best-fitting AR model (table 1) and PRCF plots (electronic supplementary material, figure S2.4). For Warnsborn, two- to three-order feedback structures were the best fit, while for the short time series of the Hoge Veluwe there was only evidence for one-order feedback. Feedback structures could replicate the cycling pattern observed in the raw data (see electronic supplementary material, figure S2.5, for an example of AR model fit for the Oosterhout area).

**Table 1.** AICc scores of autoregressive (AR) models with varying feedback orders**.** AR model selection based on AICc scores was performed on ln-transformed female winter moth densities per area, corrected for trapping effort. The best-fitting AR models (scores in italics) never exceeded four orders.

feedback order	1	2	3	4	5
Doorwerth	61.83	62.23	63.73	*60.60*	Inf
Hoge Veluwe	*46.48*	47.95	49.96	52.43	57.29
Oosterhout	78.13	79.18	77.21	*73.60*	75.35
Warnsborn	63.93	63.36	*62.73*	65.47	65.30

**Figure 6.**
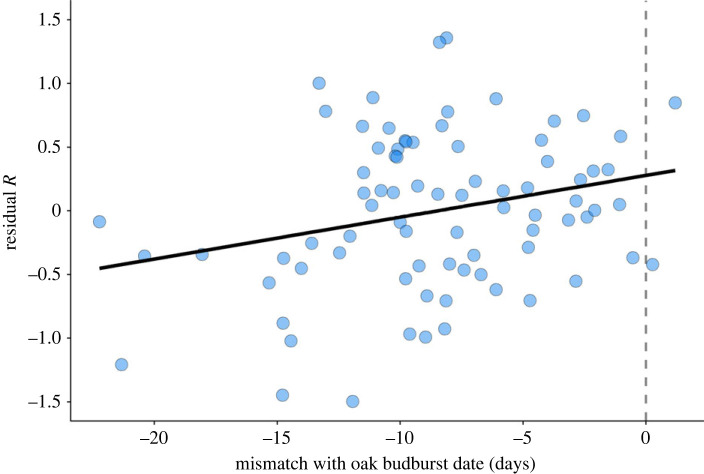
Effect of population phenological mismatch on ln-transformed realized *per capita* population growth rates (*R*) of female winter moths, corrected for cyclicity and area effects. Mismatch is expressed as days relative to oak budburst at day 0 (dashed line). Points are residuals from a model including all parameters of the final population model except for mean phenological mismatch (table 2), with the line showing the effect of phenological mismatch on these residuals. Population growth rates increased by 3.68% ± 1.52% for every day that winter moths hatched closer to budburst (i.e. for every one day decrease in mismatch magnitude, estimate = 0.04 ± 0.02 on *R*, *p* = 0.02, table 2).

We found a significant effect of phenological mismatch on the realized *per capita* growth rate of female winter moth densities (estimate = 0.04 ± 0.02, *p* = 0.02; table 2). The four feedback structures together explained 36% of the variance in growth rates, and their effects on growth rate did not differ between areas (feedback structure–area interaction effects *p* > 0.05; electronic supplementary material, table S2.1). Phenological mismatch explained an additional 6% of the variance in growth rate, with population growth rates increasing by 3.68% ± 1.52% for every day that winter moth eggs hatched closer to budburst (i.e. for every one day decrease in mismatch magnitude; figure 6). The average number of eggs per female in the previous year or the standard deviation in mismatch did not significantly explain variation in population growth rates and were removed from the final model (*p* > 0.05; electronic supplementary material, table S2.1). Mean mismatch remained significant in the full model and throughout the model simplification process when including all years (sample size ≥ 77; electronic supplementary material, table S2.2).

**Table 2.** Estimates and ANOVA results for final linear model of female winter moth realized *per capita* growth rates (*R*). The final model included four feedback structures [ln(N_t−1-4_)], as well as the mean mismatch and area as fixed effects. For each model parameter, the estimate for the log-transformed *per capita* growth rates, estimate standard error, *p*-value, estimate for the back-transformed *per capita* growth rates (effect size in %), and the proportion of variance explained (*ω*^2^ = omega squared) are shown. Significant *p*-values are in italics (*p* < 0.05).

model parameters	estimate	s.e.	*p*-value	effect size (%)	proportion of variance explained (*ω*^2^)
ln(N_t−1_)	−0.11	0.12	0.35	−10.24	0.13
ln(N_t−2_)	−0.15	0.14	0.31	−13.89	0.12
ln(N_t−3_)	0.02	0.13	0.88	1.86	0.05
ln(N_t−4_)	−0.28	0.10	*0.005*	−24.26	0.06
mean mismatch	0.04	0.02	*0.02*	3.68	0.08
area:			0.10	—	0.04
Doorwerth	1.49	0.25	—	—	—
Hoge Veluwe	1.33	0.22	—	—	—
Oosterhout	1.60	0.18	—	—	—
Warnsborn	1.09	0.20	—	—	—

## 4. Discussion

Strong selection from climate change-induced phenological mismatch can have consequences for population viability [4,5]. Over the last 20 years, Dutch winter moth populations have experienced strong mismatches between the timing of egg hatching and the timing of their food source, oak budburst, resulting in their genetic adaptation to climate change [17]. However, the exact fitness consequences of mismatch and their effects on winter moth population growth remained unclear. In this study, we carried out a phenological mismatch experiment to characterize the fitness consequences of mistimed hatching. We found that the relative fitness curve for mismatch was asymmetrical, with severe mortality rates when eggs hatched before the occurrence of budburst and reduced pupation weight when eggs hatched several days after budburst. This was reflected in the winter moth population model we constructed, which showed that mismatch had significantly influenced realized *per capita* population growth rates in the last 25 years.

### (a) Strong individual fitness consequences of phenological mismatch

The fitness curve obtained with the phenological mismatch experiment showed that fitness peaked for caterpillars that hatched two days after the date of oak budburst (figure 4). This was somewhat unexpected, as theory and previous work have placed the fitness peak on the day of budburst [21,30]. However, the relative fitness curve previously constructed for the winter moth was based only on extreme mismatches (±5 days [21]), and we obtained very similar fitness measures for these extreme mismatches in the experiment performed here (figure 2). Nevertheless, our fitness measures were taken from caterpillars that were individually housed and provided with one portion of food per feeding round. In the field, the fitness peak might be somewhat different as the fitness consequences of phenological mismatch could be further determined by interspecific competition [20] and the possibility of dispersal within-tree, or to neighbouring trees or shrubs to find alternative food sources, as there is both within- and between-tree variation in budburst date (electronic supplementary material, figure S1.1) [25] and winter moths are able to switch host plant if necessary [39].

Our day-to-day fitness measures combined into a phenological mismatch fitness curve that is asymmetrical. This asymmetrical fitness curve could mean that the costs of hatching too early in terms of high mortality are more severe than the lowered pupation weight when hatching too late. If the fitness peak for egg hatching is indeed located at 2 days after the date of budburst, this might indicate selection to evolve away from the steepest decline in fitness [4,40]. This decline for hatching too early might be even steeper as we also observed that larval development time was affected by phenological mismatch, with the shortest development times for individuals that hatched 1–2 days after budburst. Shorter development times could decrease mortality risk in the caterpillar stage through decreased exposure time to, for example, predation or adverse weather conditions [41]. However, we did not incorporate this development time variation in the relative fitness curve, as its contribution to winter moth relative fitness is unclear [29,30] and it seems to mostly mirror the effects on survival (figure 2). Lastly, it should be taken into account that we used pupation weight as a proxy for fecundity. Pupation weight was positively linearly related to adult weight as well as clutch size in winter moths raised in our laboratory (electronic supplementary material, figures S1.3 and S1.4), indicating that it is a good proxy for fecundity. If the relationship between pupation weight and clutch size is truly linear then our relative fitness curve should thus have properly accounted for the fecundity cost of hatching too late. Nevertheless, mortality during the pupal stage could also change the shape of the fitness curve as pupal weight can influence the probability of survival until adulthood [22], which we could unfortunately not measure due to the major fungal infection in the pupae.

Unexpectedly, pupal weight was affected by the photoperiod treatment caterpillars received. Caterpillars that received a constant photoperiod of early season day lengths had higher pupation weights compared to caterpillars from the naturally changing photoperiod treatment (figure 2). It could be that caterpillars in the constant photoperiod treatment anticipated a longer duration of pupation, as early season day lengths increase pupal development time in winter moths [24]. The few pupae from the constant photoperiod treatment that were not affected by the fungal infection indeed tended to emerge later as adults than pupae from the changing photoperiod treatment (electronic supplementary material, figure S1.2a). However, we believe this result is more likely due to daylight being a constraining factor. Caterpillars might feed more in dark conditions to avoid predation or they might have reduced metabolic costs due to shorter exposure to daylight [41], explaining why the caterpillars in the constant photoperiod treatment, that overall experienced fewer hours of daylight, were able to accumulate more weight.

### (b) Phenological mismatch impacted population density in the wild

Although larval mortality due to phenological mismatch is typical for winter moth populations, even before climate change occurred [15,22], strong phenological mismatch induced by global change could exacerbate this pre-existing phenological mismatch to detrimental mortality rates [42]. This could explain why we found a significant effect of mismatch on winter moth population growth rates in the last 25 years. As expected, mismatch explained only part of the variance in population growth. Nevertheless, we found a substantial effect size of mismatch with a 3.68% increase in population growth rates for every day that winter moth eggs hatched closer to budburst. In other words, the strong observed mismatches, with in some years eggs hatching 10 to 20 days before oak budburst, were estimated to have had a substantial impact on population growth (figure 6).

Our analysis suggests that winter moth populations have managed to persist at least at two out of the four investigated locations, as population cycling has continued with no obvious changes in cycle amplitude (figure 5: locations Doorwerth and Oosterhout). Thus, populations persisted despite strong individual fitness consequences of hatching too early (figure 4), continued average population mismatches in the last 25 years (figure 5), and associated decreased population growth rates (figure 6). Several ecological and life-history characteristics of the winter moth could have been important to prevent population extinction during the adaptation process to climate change, which led to a decrease in mismatch over time [17]. Firstly, winter moths caterpillars are able to disperse and use alternative food sources, which could modify the fitness curve as discussed above. Secondly, although we found no effect of mismatch variance on population growth rates in our analysis, variance in mismatch both between and within clutches could have helped winter moth populations to survive. For example, winter moths have a form of bet-hedging: within-clutch variation in timing of egg hatching varies markedly between years, with eggs laid by the same mother at times hatching over a range of multiple weeks (hatching ranges 0–45 days, median = 11 days per clutch in our long-term data, not shown). Similarly, there can be marked variation in fecundity among individuals that survived spring [43], allowing some individuals that hatched on the fitness peak to maximize fecundity. This observation has led to the idea that winter moths might have a form of evolved asynchrony, with many individuals dying from hatching too early, but the few that do survive benefitting from high fecundity [42]. These forms of risk spreading and bet-hedging could buffer populations against the consequences of extreme phenological mismatch [44,45], ensuring that (1) many females pass on at least some of their genetic material to the next generation (within-clutch variation) and that (2) population size is maintained or can recover via a few ‘winner moths' (between-clutch variation) [42].

At two of our locations, winter moth population cycling showed signs of collapse (figure 5: locations Hoge Veluwe and Warnsborn). Phenological mismatch at these two locations has been extreme even in recent years. For example, in 2017 and 2021, the late part of the winter was unusually hot followed by cold spring months, causing winter moths to advance their timing of egg hatching as egg development rate is especially sensitive to temperature from late January onwards [46], while trees probably delayed their budburst due to a different time window of temperature sensitivity [45,47]. This led to a severe mismatch between winter moth egg hatching and oak budburst of more than 15 days (figure 5). Such radical changes in weather conditions have the potential to alter density-dependent regulation of population dynamics. For example, dampening of cyclic population dynamics has been observed for four European vole species since the 1980s likely due to climatic changes [48]. Phenological mismatches can similarly impact cyclic population dynamics: in great Artic charr (*Salvelinus umbla*) predator–prey cycles have collapsed in recent years, which is thought to be due to climate change-induced phenological mismatches at two stages of their life cycle [49]. Future monitoring of these winter moth populations will indicate whether they are truly collapsing.

The observed differences in population cyclicity between the four locations could indicate that they are distinct winter moth populations. Especially, the population dynamics at the Oosterhout location showed marked differences, with a shorter cycle period that was out of sync with the other locations (figure 5). This could be related to differences in forest structure and soil characteristics as Oosterhout is located at the other side of the river Rhine compared to the other three locations (figure 1). This was, for example, the case in the gypsy moth (*Lymantria dispar*), where the frequency of population cycles differed between forest types [50]. Furthermore, migration in the winter moth is generally assumed to be minimal as females are flightless and the flight distance of males is thought to be only 1 km, which could drive local adaptation [51,52].

## 5. Conclusion

Here we showed evidence that selection has influenced population demography in the winter moth. We showed that strong phenological mismatches can lead to severe fitness consequences, especially when winter moth eggs hatched too early (figure 4). Furthermore, strong climate change-induced mismatch in the field significantly influenced population growth rates with a substantial effect size (figure 6). The winter moth's genetic adaptation to climate change might have helped some of the investigated populations to persist in the face of extreme phenological mismatch, while other populations might have started to collapse (figure 5). Future work into the eco-evolutionary dynamics of the winter moth should investigate how evolution influenced their population demography. Vice versa, population demography could have influenced evolution [6]. Further investigation into whether the winter moth's cyclic population dynamics have influenced their genetic adaptation to climate change could give important insights into what determines the evolutionary potential of wild populations.

## Data Availability

Data are available from the Dryad Digital Repository: https://doi.org/10.5061/dryad.m905qfv5p [36]. Analysis scripts can also be found on GitHub: https://github.com/NEvanDis/WM_fitness. Additional information is provided in electronic supplementary material [53].
